# Cross-sectional study of hepatitis B virus infection in rural communities, Central African Republic

**DOI:** 10.1186/1471-2334-13-286

**Published:** 2013-06-24

**Authors:** Narcisse P Komas, Ulrich Vickos, Judith M Hübschen, Aubin Béré, Alexandre Manirakiza, Claude P Muller, Alain Le Faou

**Affiliations:** 1 Viral Hepatitis Laboratory, Institut Pasteur de Bangui, PO Box 923, Bangui, Central African Republic; 2 Institute of Immunology, Centre de Recherche Publique de la Santé / Laboratoire National de Santé, Luxembourg, Luxembourg; 3 Epidemiology service, Institut Pasteur de Bangui, Bangui, Central African Republic; 4 New affiliation: EA 3452 CITHEFOR ; Faculté de Pharmacie, Université de Lorraine, Nancy, France

**Keywords:** Hepatitis B virus, HBV, Central African Republic, HBV risk factors, HBV prevalence, Rural community, HBV genotyping

## Abstract

**Background:**

As most data on hepatitis in resource-poor countries relate to urban communities, surveys in the rural environment are necessary to determine the ‘true’ prevalence of these viral infections. We undertook a survey to determine the prevalence of hepatitis B virus (HBV) infection in an apparently healthy rural population in the Central African Republic (CAR).

**Methods:**

The cross-sectional study was based on dried blood spots (DBS) from 273 people recruited in four prefectures (Lobaye, Nana-Mambéré, Ouham and Ouaka). Eluates from DBS were tested with commercial ELISA kits to detect markers of HBV infection. DBS were directly used for DNA extraction, followed by PCR and genotyping based on *preS*/*S* gene sequences.

**Results:**

The overall prevalence of HBc antibodies was 27.1% (Lobaye 29%, Nana-Mambéré 28%, Ouaka 29% and Ouham 23%) and that of HBsAg was 10.6% (Lobaye 9%, Nana-Mambéré 9%, Ouaka 19% and Ouham 8%), with no statistically significant difference among the surveyed communities. Nineteen sequences obtained from 74 anti-HBc-positive patients all belonged to genotype E. Risk factor analysis of HBV infection pointed to sexual transmission of the virus.

**Conclusion:**

The prevalence of HBV is high in rural communities in the CAR and comparable to that observed in urban areas. In addition, genotype E is prevalent in these areas. These findings underline the importance of instituting a programme of active HBV surveillance and vaccination of the population.

## Background

Despite the availability of an effective vaccine, infection with hepatitis B virus (HBV) remains a major worldwide health problem: over 2 billion people have been in contact with the virus, and there are 400 million chronic carriers and 1 million deaths per year [1]. It is hyperendemic in sub-Saharan Africa and Asia [2-5]. HBV is the type species of the *Hepadnaviridae* family and is categorized into eight genotypes (A to H) on the basis of a divergence of more than 8% in the entire nucleotide sequence of the viral DNA. Recently, two additional genotypes, I and J, were tentatively proposed [6,7].

There are several routes of HBV transmission in sub-Saharan Africa. The highest prevalence is reported in 20–40-year-olds, and horizontal transmission in early life, as a consequence of close family contact, is the most common route of infection [8,9]. In the Central African Republic (CAR), E is the prevalent genotype among HBV-infected patients, although genotypes A1, D4 and a genotype E and D recombinant have also been reported [10].

Most studies of the seroprevalence of HBV in the CAR have been conducted in urban areas, mainly Bangui [10-12], with only a few in rural areas [13,14]. In order to obtain a more precise idea of the impact of HBV infection in rural communities, we performed a survey in four prefectures of the country.

## Methods

### Study population

A cross-sectional study was carried out between November 2007 and April 2008. On the basis of an estimated HBsAg prevalence of 13% and a precision of 4%, we calculated that a minimum of 271 patients were needed. Hence, dried blood spots (DBS) were collected as previously described [11] from 273 apparently healthy individuals in the prefectures of Ouaka (n = 48), Ouham (n = 75), Nana Mambéré (n = 75) and Lobaye (n = 75). The inhabitants of the four prefectures represent one tenth of the rural population of the CAR. The area selected for sampling in each prefecture was representative of at least half (Lobaye), a third (Nana-Mambéré and Ouaka) and a quarter (Ouham) of the total population. The people living in the study areas were informed of the purpose of the study well before sample collection. Participants were recruited on a voluntary basis. To avoid collecting blood from several members of the same family, donors were selected randomly from among volunteers in each family and from among all those free of symptoms of hepatitis. Most of the participants were illiterate, and several socio-professional groups were represented. Once air-dried, the filter papers were sealed in plastic bags, taken to the laboratory at the Institut Pasteur de Bangui and stored at -20°C with a desiccant until testing.

A questionnaire on socio-demographic characteristics, such as gender, age, place of residence, education, marital status, parity, socioeconomic level and sexual practices, was completed for identification of risk factors for HBV infection. Each participant or his or her parents signed an informed consent form and was informed about the serology results. The study protocol was approved by the Scientific Committee of the Faculty of Health Sciences of the University of Bangui, CAR.

### Serological tests

Blood was eluted from DBS as previously described [11]. DBS were screened with the HBsAg Version 3, anti-HBc (total) and anti-HBc (IgM) antibody kits (Abbott-Murex Biotech Ltd, Dartford, Kent, United Kingdom). Serological tests were performed and interpreted according to the manufacturer’s recommendations.

### Detection of HBV DNA and HBV genotyping

DNA was extracted from the DBS with the QIAamp DNA Blood Mini Kit (Qiagen, Venlo, Netherlands) according to the manufacturer’s protocol. The extracted DNA was used for PCR amplification and sequencing as previously described [15]. Partial sequences covering the *preS* and *S* gene regions were aligned and compared with SeqScape v2.5 (Applied Biosystems, Nieuwerkerk, Netherlands) and BioEdit version 7.0.9.0 [16]. Phylogenetic trees were constructed with MEGA4 software [17] and the neighbour-joining and Kimura 2-parameter methods. Sequences representative of known genotypes and sub-genotypes and the most similar previously published sequences identified by BLAST were downloaded from GenBank and included in the analysis. The nucleotide sequence data reported in this paper are available under GenBank accession numbers JQ972779–JQ972830.

### Statistical analysis

Stata 11.0 software was used to determine the prevalence rates of HBV; the Fisher exact test was used to compare variables and the association between HBV positivity and risk factors such as marital status, years since first sexual intercourse, use of condoms, number of sexual partners, socio-professional activity and previous risk behaviour. These risk factors of HBV infection were assessed by estimating odds ratios. Confidence intervals were calculated at 95%. Statistical significance was assessed at *p* < 0.05.

## Results

### Serology and geographical distribution of hepatitis B virus in the four prefectures

In the tested population, comprising 173 (63.7%) females and 100 (36.6%) males aged 2–67 years (mean, 27 years, SD ± 15), 74 people (27.1%) had anti-HBc antibodies, indicating that a high proportion of these rural populations had been exposed to HBV. The population prevalence was estimated as 24.2% by standardizing the seroprevalence results obtained according to the gender and age group distribution of the CAR population. HBsAg was found in 29 (10.6%) donors, and 7 (24.1%) had been recently infected or presented with acute viral hepatitis B (anti-HBc IgM antibody positive). Five were aged 17–25 years and two were more than 25 years old. Forty-five (16.5%) individuals were positive for anti-HBc but negative for HBsAg.

**Figure 1:**
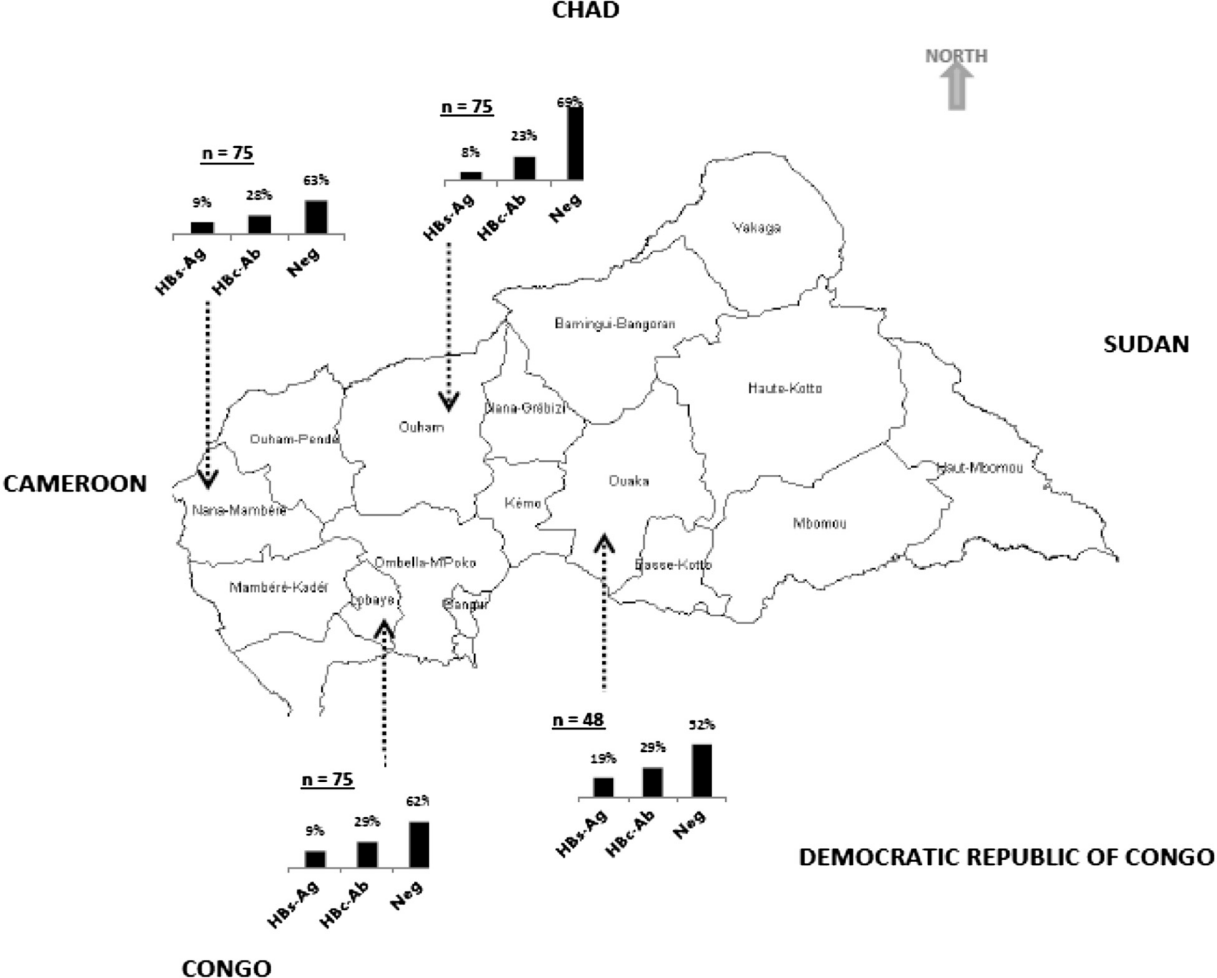
Geographical location of the four surveyed prefectures and their HBsAg and HBc antibody prevalence.

The prevalence of HBV infection was higher in males than in females over 16 years of age (*p* = 0.03, Table 1). Although no statistically significant differences in the overall HBV and HBsAg prevalence in the four prefectures were observed, a higher HBsAg rate was found in Ouaka than in the other three locations (Figure 1).

**Table 1:** HBV positivity by sex and age among 273 donors in rural regions of four prefectures of the Central African Republic

**Age (years)**	**Male**		**Female**		**Total**	
	**n positive (Total)**	% of *HBV+ (95% CI)	**n positive (Total)**	% of *HBV+ (95% CI)	**n positive (Total)**	% *HBV+(95% CI)
0–15	5 (29)	17.8 (3.5–31.0)	5 (28)	17.8 (3.7–32.0)	10 (57)	17.5 (8.4–32.3)
16–25	18 (40)	45.0 (29.6–60.4)	20 (58)	34.5 (22.2–46.7)	38 (98)	38.8 (27.4–53.2)
> 25	8 (31)	25.8 (10.4–41.2)	18 (87)	20.7 (12.2–29.2)	26 (18)	22.0 (14.4–32.3)
All	31 (100)	31.0 (21.1–44.0)	43 (173)	25.0 (18.0–33.5)	74 (273)	27.1** (21.3–34.0)

*At least one marker (HBsAg or anti-HBc antibody).

**The estimated population prevalence after taking into account the gender and age group distribution of the CAR is 24.2%.

### Risk factors for HBV infection

No statistically significant association was found between HBV infection and socio-demographic characteristics. However, monogamous males had a slightly higher HBV prevalence (40% or 16/40) than single or polygamic males (24% or 12/49) (Table 2) (*p* value = 0.18). Sexual behavior (years since first sexual intercourse, use of condoms and number of sexual partners) was only moderately associated with HBV infection. The only profession associated with HBV infection was small trader (Table 2). Although the HBsAg prevalence seems to be higher in the Ouaka prefecture than in the three other prefectures no particular predisposing risk factors were identified, maybe because of the limited number of positive people.

**Table 2:** Risk factors for HBV infection

**Variable**	**n**	*HBV+ (%)	**Odds ratio**	**95% CI**
Marital status				
○ Male				
• Monogamous marriage	40	16 (40.0)	1.00	
• Single	49	12 (24.5)	0.51	0.20–1.27
• Polygamous marriage	11	03 (27.0)	0.56	0.12–2.44
○ Female				
• Monogamous marriage	81	22 (27.1)	1.00	
• Single	73	16 (22.0)	0.75	0.35–1.57
• Polygamous marriage	19	05 (26.3)	0.95	0.30–2.97
Years since first sexual intercourse				
○ ≤ 10	99	26 (26.3)	1.00	
○ 11–20	48	17 (35.4)	1.53	0.73–3.23
○ 21–30	31	11 (35.5)	1.54	0.65–3.65
○ > 30	28	06 (21.4)	0.76	0.27–2.09
Use of condoms				
○ Yes	19	06 (31.6)	1.00	
○ No	227	64 (28.2)	1.12	0.40–3.10
Number of sexual partners				
○ One	6	02 (33.3)	1	
○ Two or more	102	32 (31.4)	1.20	0.21–6.83
Socio-professional activity				
○ Civil servant	24	05 (20.8)	1.00	
○ Small trader	10	05 (50.0)	3.80	0.78–18.50
○ Farmer	63	19 (30.2)	1.64	0.53–5.04
○ Student	74	22 (29.7)	1.60	0.53–4.84
○ Unemployed	28	05 (17.8)	0.80	0.20–3.28
○ Others**	65	18 (27.7)	1.45	0.47–4.48
Previous risk behaviour				
○ None	71	22 (31.0)	1.00	
○ Scarification	169	44 (26.0)	0.78	0.42–1.44
○ Surgery	47	12 (25.5)	0.76	0.33–1.74
○ Blood transfusion	30	04 (13.3)	0.34	0.10–1.10
○ Tattooing	31	09 (29.0)	0.91	0.36–2.29
○ Dental surgery	33	10 (30.3)	0.96	0.39–2.37

*At least one marker (HBsAg or anti-HBc antibodies).

**Others: carpenter, apprentice, driver, night watchman, craftsman.

### Sequence analysis and genotyping

**Figure 2:**
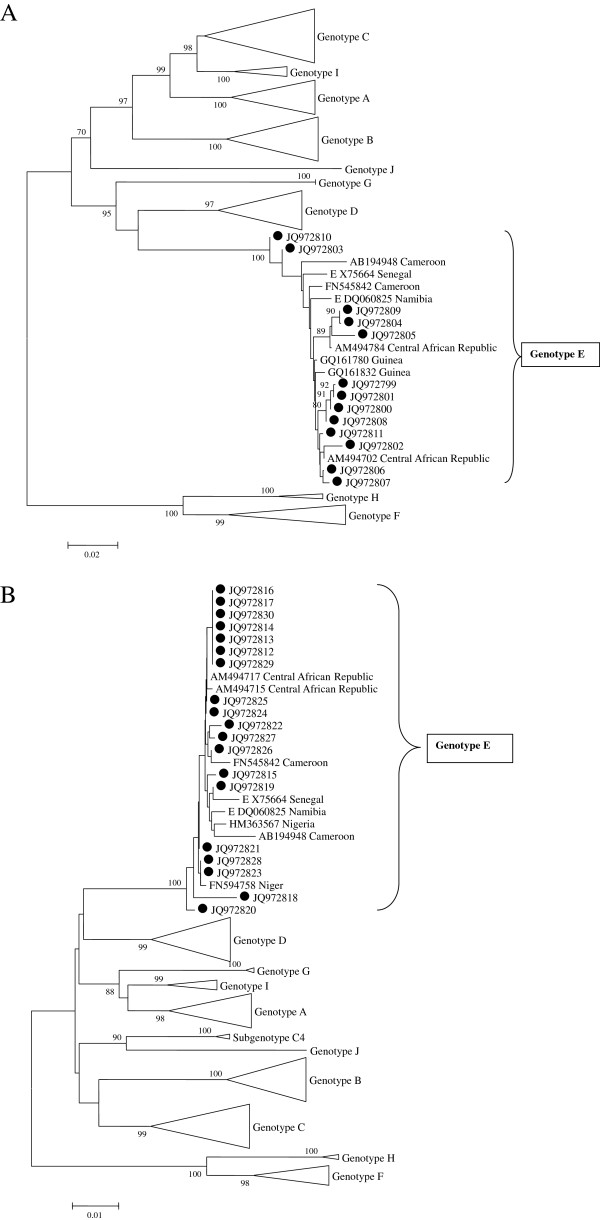
**Phylogenetic tree based on 763 nucleotides of the *preS *(A) (13 strains) and 884 nucleotides of the *S *(B) (19 strains) gene regions of HBV strains from the Central African Republic (marked with a black dot) and reference strains of recognized and proposed genotypes and closest BLAST fits; GenBank accession numbers are indicated in the tree.** Only bootstrap values (n = 1000) ≥ 70 are shown.

Sequence information was obtained for 19 of the 74 anti-HBc antibody-positive samples tested, with 14 sequences from HBsAg-positive samples and 5 from people positive only for antibodies to HBc. All the sequences belonged to genotype E and were similar to strains from CAR, Cameroon, Guinea and Niger (Figures 2A and 2B).

## Discussion

The overall prevalence of HBV detected in the present study was slightly higher than that reported in blood donors in Burkina Faso (14.96%) [18], lower than those reported in pregnant women in the United Republic of Tanzania (56.2%) [19] and in Ghana (67.1%) [20], in a cohort of students in Bangui (42.3%) [11], in blood donors in Cameroon (86.8%) and Sudan (36%) [21,22] and in the staff of a public hospital in Luanda (79.7%) [23]. The diversity of the observed prevalence is probably due to the diversity of patients and population groups and differences in sampling method, test kit sensitivity and specificity or differences between rural areas and large cities. In large cities, population movement and a permissive lifestyle increase the risk for exposure to this disease.

Dried blood spots are now commonly used for serology and molecular biology testing. The only problem encountered so far is a slightly lower sensitivity, although reliable results are obtained. The technique permits surveys to be conducted in remote areas, as it requires little equipment, the cost of sampling is low, and it is widely accepted as it is almost painless [11].

A better estimate of the prevalence of HBV infection in rural areas throughout the country would have been obtained if we had surveyed all 16 prefectures; however, this was not possible because of the absence of practicable roads and wide insecurity. Nevertheless, the prevalences of HBV in the four prefectures studied (Lobaye 29%, Nana-Mambéré 28%, Ouaka 29% and Ouham 23%) are similar and lower than the only previous prevalence data published more than two decades ago in the prefecture of Ouham-Pendé (61% in 1984 and 48% in 1988) [13]. Similarly, the HBsAg prevalence was lower than in Ouham-Pendé (20.9%) [14] in all prefectures except for Ouaka (19%, Figure 1). The high HBsAg prevalence in Ouaka prefecture was not statistically different from that in the other three prefectures, probably because of the relatively small number of people investigated. Individuals positive for antibodies against HBc but HBsAg negative were found commonly (16.5%), and viral DNA was detected in five. Thus, the number of patients with active HBV infection is underestimated if only HBsAg prevalence is considered. Inability to detect HBsAg may be due to a mutation (G145R for example, which abolishes HBsAg specificity); however, such mutants are unlikely to be selected by vaccination [24], which in the CAR is given only to young children within the expanded programme of immunization (EPI) . A high prevalence of HBV DNA in the absence of HBsAg was previously described among students in Benin, which was not explained by mutations in the *S* gene sequence [25], and has also been observed previously in the CAR [10]. A certain characteristic of the population that favours occult infection is a possibility and requires additional investigation [26]. Occult infections are often overlooked, as these patients are considered uninfected on the basis of HBsAg test results. As a consequence, a diagnosis of chronic hepatitis B should systematically include screening for HBV DNA when antibodies to HBc are present and HBsAg is not.

Analysis of the socio-demographic data did not reveal any significant risk factor for acquiring HBV infection in rural areas of the CAR. Nevertheless, small traders were more often affected than people in other professions (50%); sexual practices (30–40%), tattooing (29%), blood transfusion (13%) and dental surgery (10%) were other possible risk factors. These percentages were much lower than in a cohort of students in Bangui [11] (sexual practices, 38.2–53.3%; tattooing, 42.4%; blood transfusion, 39.4%; dental surgery; 38%). It is possible that other unknown or unstudied risk factors contribute to the transmission of HBV in rural CAR, and large-scale studies should be performed, including more risk factors.

In contrast to other studies [4,21,23] that showed a certain diversity of HBV genotypes in Central African countries, this study confirms that genotype E is highly predominant, not only in Bangui [10] but also in rural areas of the CAR, supporting the hypothesis that this country is part of the vast genotype E crescent, spanning Africa from Senegal to Angola [25]. Characterization of HBV nucleotide sequences from a large sampling in rural CAR should be undertaken to better evaluate the genetic variability of this virus in these areas.

## Conclusion

HBV infection is a major health issue in the CAR, as shown by high prevalence rates. The prevalence in the four prefectures is close to that in Bangui, and only genotype E sequences were found. This high prevalence confirms that HBV is responsible for high rates of morbidity and mortality in CAR. The results underline the necessity for adult vaccination against hepatitis B in addition to HBV vaccination included in the EPI since November 2008.

## Abbreviations

Anti-HBc Ab: Anti-hepatitis B core antibody; CAR: Central African Republic; DBS: Dried blood spots; EPI: Expanded Programme for Immunization; HBsAg: Hepatitis B surface antigen; HBV: Hepatitis B virus; PCR: Polymerase chain reaction.

## Competing interests

The authors have neither commercial interest in the present study nor any conflict of interest. The Institut Pasteur de Bangui and the Ministry of Foreign Affairs in Luxembourg supported the study financially.

## Authors’ contributions

NPK designed the study and directed it, supervised all the field activities, analysed and interpreted the data and wrote the manuscript. UV helped to supervise the field activities, contributed to the acquisition of data by preparing the questionnaire and by participating in the collection of dried blood spots. JMH analysed the molecular data and contributed to writing the manuscript. AB contributed to the acquisition of data by performing ELISA tests. AM prepared the questionnaire and analysed and interpreted the epidemiological data. CPM and ALF contributed to the analysis and interpretation of the data and to writing the manuscript. All the authors approved the final version of the manuscript.

## Pre-publication history

The pre-publication history for this paper can be accessed here:

http://www.biomedcentral.com/1471-2334/13/286/prepub
